# A fingerprint pair analysis of hERG inhibition data

**DOI:** 10.1186/1752-153X-7-167

**Published:** 2013-10-21

**Authors:** Clayton Springer, Katherine L Sokolnicki

**Affiliations:** 1 Novartis Institutes for BioMedical Research, 100 Technology Square, Cambridge, MA 02139, USA

**Keywords:** Fingerprint pairs, hERG, Molecular pair, Hydroxyl, Molecular matched pairs, Cliff pairs, Extended connectivity fingerprints (ECFP)

## Abstract

**Background:**

Drugs that bind to the human Ether-a-go-go Related Gene (hERG) potassium channel and block its ion conduction can lead to Torsade de Pointes (TdP), a fatal ventricular arrhythmia. Thus, compounds are screened for hERG inhibition in the drug development process; those found to be active face a difficult road to approval. Knowing which structural transformations reduce hERG binding would be helpful in the lead optimization phase of drug discovery.

**Results:**

To identify such transformations, we carried out a comprehensive analysis of all approximately 33,000 compound pairs in the Novartis internal database which have IC_50_ values in the dofetilide displacement assay. Most molecular transformations have only a single example in the data set; however, a few dozen transformations have sufficient numbers for statistical analysis.

**Conclusions:**

We observe that transformations which increased polarity (for example adding an oxygen, or an *sp*^*2*^ nitrogen), decreased lipophilicity (removing carbons), or decreased positive charge consistently reduced hERG inhibition between 3- and 10-fold. The largest observed reduction in hERG was from a transformation from imidazole to methyl tetrazole. We also observe that some changes in aromatic ring substituents (for example hydrogen to methoxy) can also reduce hERG binding in vitro.

## Background

Inhibition of the human Ether-a-go-go Related Gene (hERG) channel can be a limiting toxicity for drug candidates. The hERG channel regulates transmembrane movement of potassium ions and is a major contributor to the repolarization phase of the cardiomyocyte action potential in the heart [1]. Inhibition of the hERG channel causes lengthening of the cardic QT interval, which can lead to Torsade de Pointes (TdP) [2]. It was this toxicity that in 1997 led to the withdrawal of terfenadine (Seldane) [3]. Although the Redfern criteria is that the IC_50_ (half maximal inhibitory concentration in vitro) be more than 30 fold greater than the C_max_ (the maximum plasma concentration in vivo), typically during lead optimization the C_max_ (or dose) is not known [2]. However, project teams can estimate toxicity from in vitro IC_50_s. For drugs with submicromolar C_max_, an in vitro hERG inhibition IC_50_ of greater than 30 micromolar (μM) in the radio-ligand binding assay [4] is generally considered desirable; having an IC_50_ of less 10 μM is cause for concern and must be improved. The frequency and severity of hERG inhibition drives drug discovery teams to make considerable efforts in measuring, analyzing, and mitigating hERG inhibition [4].

QSAR (Quantitative structure–activity relationship) models using machine learning algorithms [5,6] are established tools for analyzing biological activity data, either with linear (linear discriminate analysis (LDA), partial least squares (PLS) [7]) or non-linear (multi-layer perceptrons [8] support vector machines (SVM), random forest, multivariate adaptive regression splines (MARS) methods. These models find several important descriptors of hERG inhibition, including AlogP or ClogP (measures of lipophilicity), the presence of two lipophilic atoms separated by 10 bonds, fluorine atom count, carbon-carbon double bonds, the presence of a hydroxyl, and partial negative surface area [7,8]. Regardless of the method chosen, the resulting mathematical model is smoother than the sharpest changes in the data [9]. This contrasts with the needs of lead optimization, in which one wants to find the smallest chemical change that makes the best similar compound. To find the sharpest changes in the data, it is molecular pair analysis rather than global QSAR models that is most applicable approach [10,11].

Matched molecular pairs analysis defines a transformation as a change at an attachment point (this may be generalized to multiple attachment points). Literature reports of analysis of pairs started in the mid-2000s [9,12,13]. Leach *et al.* aggregated their aqueous solubility, plasma protein binding and oral exposure data with pre-specified transformations [14]. In a very comprehensive analysis of GlaxoSmithKlines’s internal data, Papadatos *et al.* analyzed the effects that many matched pairs transformations had on hERG inhibition [15]. The authors analyzed those pairs for context and found several that were statistically different from the overall matched pair average and for hERG they gave details for 3 such examples.

**Figure 1:**
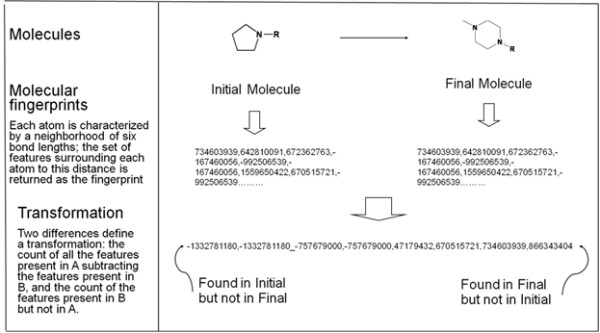
**A schematic of a transformation and its ECFP representation.** At the top of the schematic, we show the initial and final molecules, and their EFCP fingerprint representation. The last line shows the EFCP representation of the transformation is characterized by which EFCP fingerprints disappear from the initial molecule and which appear in the final molecule.

The SALI (Structure-Activity Landscape Index) approach to pairs analysis uses similarity distance (typically fingerprint based) to identify pairs [10]. While SALI may be useful for inspection of individual (also called cliff) pairs, these singleton examples lack statistical significance. To identify transformations that have a Wilcoxon consistent effect on hERG inhibition, we introduce fingerprint pairs, which are an extension of the SALI approach. Aggregating the pairs allows us to make observations about which transformations have an effect on hERG binding (see Figure 1 and Methods section). In contrast with matched pairs approaches, which aggregate the pairs by breaking at a single bond, in our approach the fingerprint pairs implicitly aggregate based on contextual information.

## Results and discussion

With a list of individual fingerprint pairs, we then collect the individual pairs into aggregates. We then make observations about the whole collection of transformations. We move onto the discussion of particular transformations where we have enough supporting examples. We conclude with some observed trends across these pairs.

### Aggregate size distribution vs. mean effect of hERG inhibition

**Figure 2:**
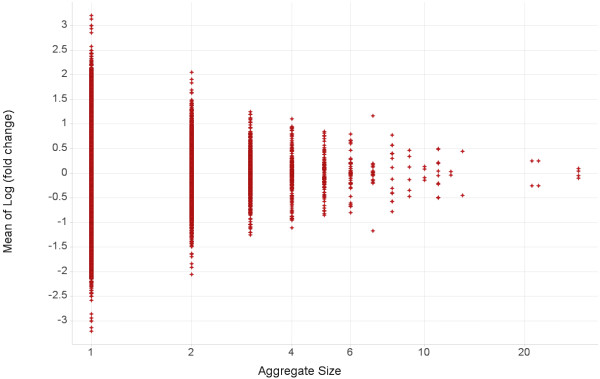
**Aggregate size vs. its mean change in log potency.** Each point is an aggregate of the same kind of transformation. The x-axis shows the numbers of examples the aggregate has and the y-axis shows the geometric average of fold change in each aggregate. See text for discussion.

After computing all the transformations in the data, we group the pairs such that those making the same chemical transformation are aggregated together. Each aggregate is summarized by its mean hERG inhibition and the number of pairs in the aggregate (Figure 2). The x-axis shows the number of examples it has and the y-axis its average change in log (hERG) inhibition. The graph’s top and bottom halves are symmetrical about the y-axis because each transformation also appears in reverse. In the reverse transformation, the initial molecule, final molecule, the sign of the difference vector, and the sign of the log (fold change) are all reversed.

To determine which aggregates have sufficient statistical power, we use the Wilcoxon distribution, since we do not assume the data are normally distributed. Most transformations probably have at least a small effect on hERG inhibition; however, for aggregates with 4 or fewer examples (that is almost all aggregates), the Wilcoxon confidence interval includes zero or no change. We discard these aggregates and proceed only with those aggregates containing five or more examples (see Methods for details).

In our data set of ~33 K aggregates, there are 112 aggregates with five or more examples; that is, 56 transformations and their reverse transformations. Each is considered individually. While the observed changes in these transformations may be explainable by various models based on SlogP, predicted ionization, and/or aromatic atom counts, such quantitative analysis is beyond the scope of this paper. However, we do note the ΔSlogP in each aggregate.

### Size distribution of aggregates follows a power law distribution

**Figure 3:**
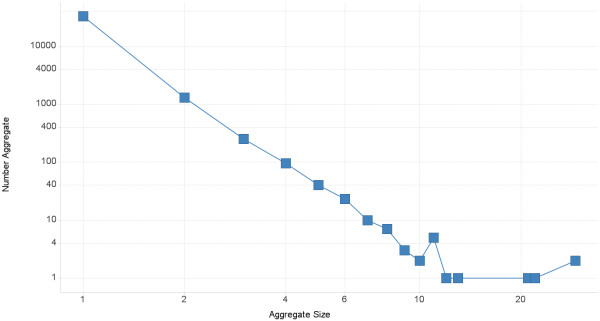
**A histogram of aggregate size.** The x-axis is aggregate size and the y-axis is the numbers of aggregates at that size in log scale at that size. Aggregate sizes follows a power law distribution. See text for discussion.

Figure 3 shows the size of aggregates versus the frequency of their occurrence in the data set. These are the examples from the top half of Figure 2 (that is, one direction of the transformation). The distribution of sizes of collected aggregates roughly follows a power law: a few transformations occur commonly, very many occur infrequently, and most transformations are only seen once. The power law distribution is also observed when 3the transformations are aggregated as matched molecular pairs. In this data set, 32,802 of the transformations are singletons (that is, they are size 1); just two aggregates have 28 examples.

### Sets of transformations

**Figure 4:**
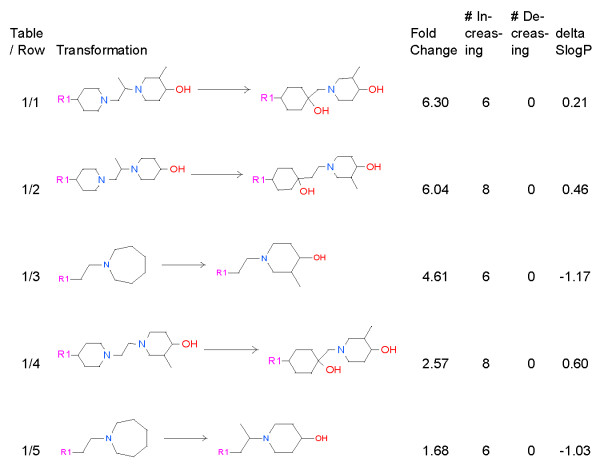
**Transformations that add oxygen and reduce hERG inhibition.** For each transformation we show the fold reduction in hERG inhibition, the number of examples that increase the IC_50_, the number that decrease the IC_50_, and change that this transformation makes in the SlogP model of logP.

As discussed above, only 56 aggregates had sufficient examples to allow for conclusions to be drawn about the chemical transformation. Of these 56, only 17 made a significant reduction in hERG inhibition. Figure 4 through 7 enumerate these transformations. In many of the transformations hERG inhibition follows the expected qualitative trends: decreasing lipophilicity, decreasing basicity, and increasing acidity all decrease the potency of hERG blockers. While our observations reflect consistent behavior seen in the database, we cannot claim that they are completely general because of the narrowness of the molecular context. Due to their proprietary nature, we cannot disclose the full structures, but the molecules in the transformations are similar to each other. Given the range of changes in hERG inhibition IC_50_s observed in our data set, improvements by 2- and 3-fold are notable.

### Adding aliphatic oxygen frequently reduces hERG inhibition

We observe that transformations that introduce a hydroxyl near an amine and changing a 7 member ring into a 6 membered ring and a methyl group reduce hERG inhibition by 2 to 6 fold (Figure 4, Rows 3 and 5). There are no transformations in which introducing a hydroxyl near an amine increases hERG binding (see Figure 4). Changing a molecule with two amines into a system with one amine and a hydroxyl group reduces hERG inhibition by 4 to 6 fold (Figure 4, Rows 1, 2 and 4), and increases SlogP/estimated lipophilicity.

### Aromatic substitutions reduce hERG inhibition

**Figure 5:**
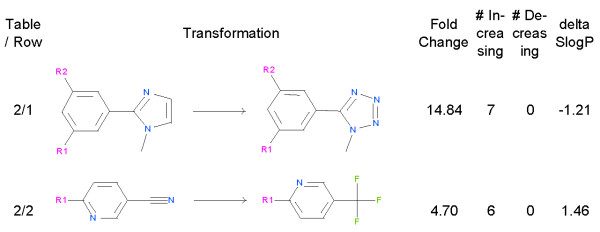
**Transformations that alter an aromatic system.** For both transformations we show the fold reduction in hERG inhibition, the number of examples that increase the IC_50_, the number that decrease the IC_50_, and change that this transformation makes in the SlogP model of logP.

Basic nitrogen atoms are the key to potent hERG blockers; however, introducing *sp2* nitrogens reduces inhibition in many transformations. Our largest observed reduction in hERG inhibition lowers hERG inhibition by 15 fold by adding *sp2* nitrogens to the slightly basic imidazole to obtain the somewhat less lipophilic methyl tetrazole (Figure 5, row 1). Changing a pyridyl nitrile to a -CF3 group reduces inhibition by 4.7 fold (Figure 5, row 2). We speculate that these reductions in hERG inhibition come from reducing lipophilicity and/or altering the energetics of pi-stacking of the inhibitor’s aromatic groups with aromatic groups in the hERG channel. However, exploring this question is beyond the scope of this paper [16].

### Changing the environment of the amine nitrogen can reduce hERG inhibition

**Figure 6:**
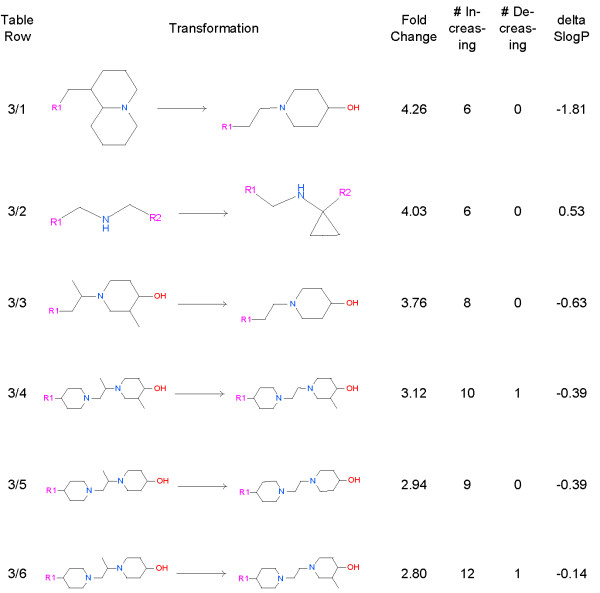
**Transformations which remove carbons or change the N environment.** For these transformations we show the fold reduction in hERG inhibition, the number of examples that increase the IC_50_, the number that decrease the IC_50_, and change that this transformation makes in the SlogP model of logP.

Transformations in Figure 6 show that removing carbons and/or changing the electronic environment around the basic nitrogen can result in a modest reduction in hERG binding. The transformation in Figure 6, Row 1 shows us that removing carbon atoms and adding a hydroxyl has a consistent and substantial effect on hERG inhibition. Row 2 shows us that adding a cyclopropyl adjacent to nitrogen reduces the hERG inhibition. It is the reduction in basicity [17] that is likely responsible for this change. Three transformations show that removing carbon atoms reduces hERG inhibition (Figure 6, rows 3, 4 and 5). The last row (Figure 6, row 6) shows that the effect is not simply reducing lipophilicity, but is also from changing the chemical environment around the nitrogen, in particular the removal of the beta carbon.

### Miscellaneous transformations that reduce lipophilicity

**Figure 7:**
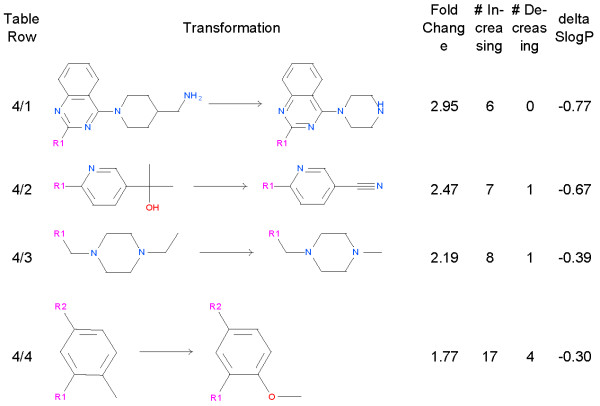
**Miscellaneous lipophilicity reducing transformations.** For these transformations we show the fold reduction in hERG inhibition, the number of examples that increase the IC_50_, the number that decrease the IC_50_, and change that this transformation makes in the SlogP model of logP.

In this Figure the transformations that reduce hERG inhibition generally reduce lipophilicity. The transformation in Figure 7, row 1 which removes 2 carbons and changes the primary nitrogen into a secondary nitrogen improves hERG by 3 fold. Going from pyridyl, gem dimethyl hydroxyl to pyridyl nitrile (Figure 7, row 2) improves hERG by 2.5 fold and reduces inhibition in 7 out of 8 examples. Going from ethyl piperazine to methyl piperazine (Figure 7, row 3) results a 2.2 fold reduction in hERG inhibition. This change may result from lipophilicity and/or pKa effects on the nitrogen. A transformation from methyl to methoxy (Figure 7, row 4) has a small effect (~1.7 fold), reducing hERG binding in 17 out of 21 examples.

### A comment on transformations not observed

The direct addition of a carboxylic acid or the removal of an amine group does not occur in any aggregate with 5 or more examples. While we would expect these transformations to make a difference in hERG inhibition, our approach also looks at context, and none of these transformations appear with enough examples to pass our statistical threshold. Other techniques are needed to observe this effect (and others) in the data.

### ΔSlogP versus Δlog (hERG) for the transformations

**Figure 8:**
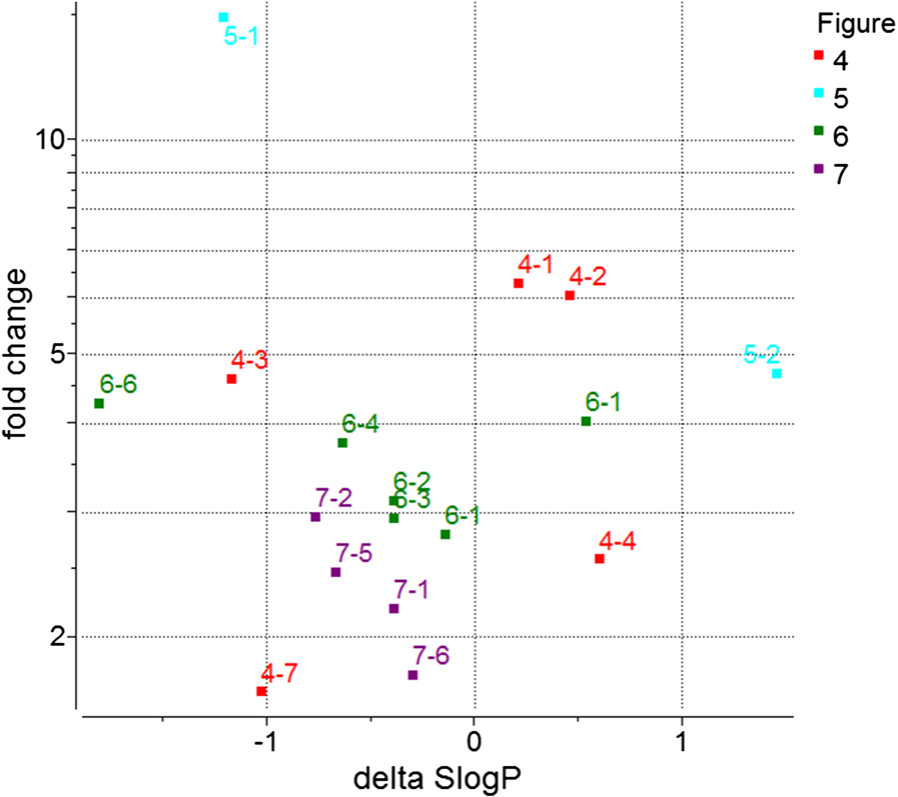
**This plot shows the ΔSlogP versus Fold Change (log scale) for the transformations.** Each point is labeled by the Figure and Row in which it appears. See text for discussion.

Although we expect SlogP to make a difference in hERG inhibition, Figure 8 shows that its effect is not determinative. It is likely confounded by ionization and as well as other factors. We observe a number of transformations that reduce hERG inhibition even while increasing the SlogP. The points in Figure 8 are labeled with the Figure and Row in which they appear.

## Conclusions

An attractive aspect of our approach, which does not use a preselected list of transformations, is that the resulting pair list is comprehensive in the data set used; that is, every transformation in the dataset is considered in the analysis. However because of the stringent statistical approach we applied most transformations are not used. One approach to extracting additional information from this dataset is to build a QSAR model on the vector fingerprint change of the transformations. Another attractive aspect of pair analysis for informing lead optimization is that the analysis is directly in the form of a change in chemical structure. Specific structural changes are revealed to reduce hERG binding, rather than indirect through descriptors like SlogP [18], PSA [19], Chi-Square [20] or BCUT [21]. In broad terms, the transformations’ effects are straightforward; for example, the removal of the basic nitrogen or the manipulation of the pKa of the basic nitrogen environment by addition or removal of nearby alkyl groups. This approach found some subtle transformations that reduce hERG binding, including adding hydroxyls, adding *sp2* nitrogens, or putting amide substitutions on aromatic rings. We expect that just as cheminformatics tools are currently applied to molecular representations to cluster, search and model molecules, these approaches can be applied to cluster, search and model transformations.

We observe that most transformations have no more than a modest change in hERG inhibition. This reinforces our impression from medicinal chemistry that hERG inhibition has a rather flat structure activity relationship: we rarely observe subtle changes in structure that result in dramatic changes in activity. Contrast this with biochemical potency, where subtle structural changes can often result in abolishing activity. Biochemical potency depends on receptors that have been designed by evolution to be sensitive to subtle changes in chemical structure (for example estrogen vs. testosterone, or epinephrine vs. norepinephrine) whereas the hERG channel has been designed to transport potassium and has had little or no evolutionary pressure to be selective against micromolar concentrations of aromatic amines.

## Methods

Dofetilide displacement measurement data [22] were extracted from the Novartis corporate database. We computed the pairwise difference in the log of measured IC_50_ for each compound pair. Pairs were excluded if both measurements were off scale in the same direction. Otherwise, off-scale measurements were treated numerically as being at the extreme end of the scale; i.e., every value >30 μM was treated as 30 μM. We characterized the chemical structure with Pipeline Pilot’s extended connectivity fingerprints (ECFP) [23]. We use the ECFP family of fingerprints because of their utility in cheminformatics applications [12,24]. The transformation between molecules was represented by the “difference fingerprint”. In the difference fingerprint we record those ECFPs that disappear from the initial molecule and that appear in the final molecule (see Figure 1). The fingerprint approach is both much faster than maximum common substructure (MCS) methods and implicitly includes some molecular context. However, advances in MCS methods have reduced the computational effort needed to calculate MCS based matched pairs [25].

We define the aggregates by the change in fingerprint: pairs are put together in the same transformation if and only if they have the same change in fingerprint. In ECFP-N fingerprints, the molecules are characterized by substructures around each atom, and the ‘N’ denotes the maximum diameter of the substructures used. Thus, in ECFP0 fingerprints these substructures are just a count of the different atoms and the fingerprint has equivalent information to the molecular formula. We did not choose N = 0 because all transformations with the same change in molecular formula would have been aggregated together, and an aggregate would contain molecular pairs that are making different chemical transformations. Using ECFP2 has similar drawbacks. We initially tried N = 4, but not all the pairs it grouped together were similar enough. After tightening our criteria one step further to N = 6, the aggregates represent the same chemical transformation. However this came at the cost of spreading the available data over more aggregates, which reduced the number of aggregates with enough examples to make statistically definitive statements.

To assess the statistical power of a particular transformation, we consider the probability that a particular distribution of either increases or decreases IC_50_ would be observed by chance alone. Our null hypothesis is that the transformation on average has no systematic effect. From the Wilcoxon, we estimate the likelihood of observing a particular distribution IC_50_s occurring by chance. In the null hypothesis, a sample size of 5 pairs which all either increase or decrease occurs 6.25% of the time. This gives us our threshold of 5 examples. For a sample size of 8 pairs, samples that have 7 increases and 1 decrease or 1 decrease and 7 increases occur (that is, has a p-value of) 5.46% of the time (see Additional file 1: Figure S1 on the paired Wilcoxon distribution).

Many biochemical assays have substantial correlation between measured IC_50_ and logP (a measure of lipophilicity). Because we lack logP measurements for many of our molecules we use a Crippen’s model of logP. In particular, we use MOE’s [26] (Chemical Computing Group, Montreal QC, Canada) implementation of that model which it calls SlogP [18]. SlogP is based on atom types, so each molecular pair in a particular aggregated transformation has the same change in SlogP.

## Abbreviations

Cmax: Maximum in vivo plasma concentration; SALI: Structure-activity landscape index; logP: Measurement of a compound’s equilibrium partitioning between octanol and water; SlogP: LogP calculator found in MOE^21^; hERG: Human ether-a-go-go related gene; IC50: Half maximal inhibitory concentration; MOE: Molecular operating environment, a software product of the Chemical Computing Group; MCS: Maximum common substructure; ECFP: Extended connectivity fingerprint; μM: Micromolar; nM: Nanomolar.

## Competing interests

We have no competing interest financial or otherwise in these techniques.

## Authors’ contributions

CS devised the research plan, supervised the work, and drafted the manuscript. KS carried out the day-to-day computational work. Both authors read and approved the final manuscript.

## Authors’ information

CS works as CADD scientist at Novartis. KS was a summer intern in his lab.

## Supplementary Material

Additional file 1: Figure S1 Shows the Wilcoxon statistical significance for the different aggregiates observed in our data set. Each symbol in the graph represents an aggregate. The x-axis shows the number of examples that increase the hERG inhibition. The y-axis shows the number of examples that decrease hERG inhibition. The aggregate is colored by its Wilicoxon p-value (all the aggregates with the same number of increasing and deceasing examples have the sample Wilcoxon p-value). The total number of paired values is given by the sum of these two thus there is no 0,0 point. For example an aggregate with 0 increases, 5 decreases (that is 5 total) has significance value of <0.05 (~0.03). For an aggregate of 8 pairs, 1 increase and 7 decreases has statistical significance. Click here for file
