# Supplementary figures and images for: A fingerprint pair analysis of hERG inhibition data
Source: Chem Cent J. 2013 Oct 21;7:167. doi: 10.1186/1752-153X-7-167 (PMC3854090; doi:10.1186/1752-153X-7-167)

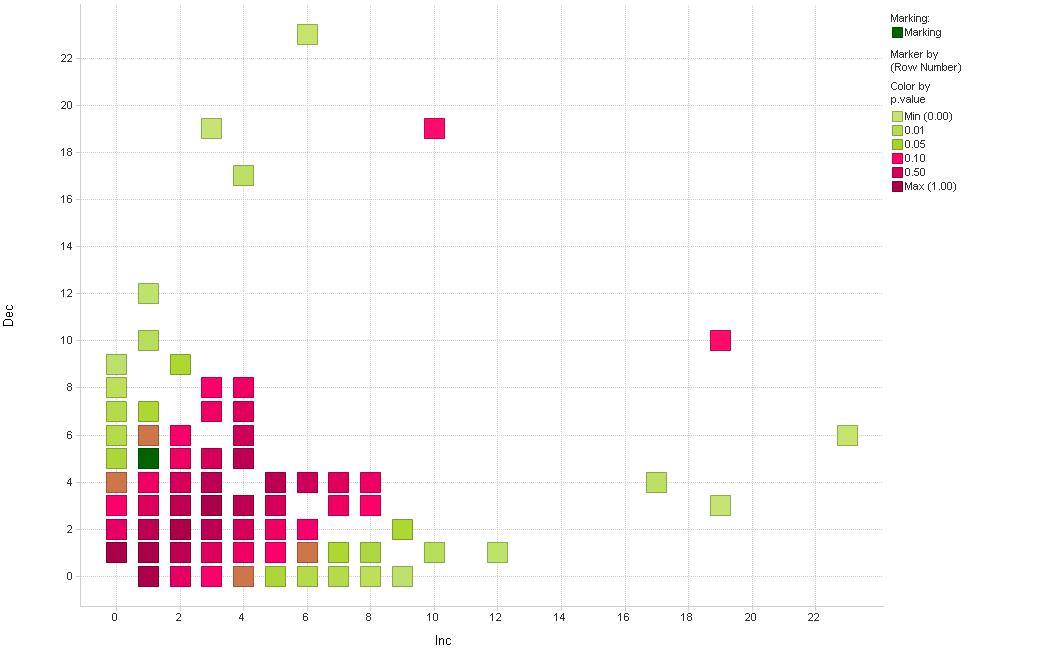

Supplement: Additional file 1: Figure S1 — Shows the Wilcoxon statistical significance for the different aggregiates observed in our data set. Each symbol in the graph represents an aggregate. The x-axis shows the number of examples that increase the hERG inhibition. The y-axis shows the number of examples that decrease hERG inhibition. The aggregate is colored by its Wilicoxon p-value (all the aggregates with the same number of increasing and deceasing examples have the sample Wilcoxon p-value). The total number of paired values is given by the sum of these two thus there is no 0,0 point. For example an aggregate with 0 increases, 5 decreases (that is 5 total) has significance value of <0.05 (~0.03). For an aggregate of 8 pairs, 1 increase and 7 decreases has statistical significance. [file 1752-153X-7-167-S1.png]
